# The Liver X Receptor Is Upregulated in Monocyte-Derived Macrophages and Modulates Inflammatory Cytokines Based on LXR*α* Polymorphism

**DOI:** 10.1155/2019/6217548

**Published:** 2019-02-28

**Authors:** Hyoun-Ah Kim, Wook-Young Baek, Mi-Hwa Han, Ju-Yang Jung, Chang-Hee Suh

**Affiliations:** Department of Rheumatology, Ajou University School of Medicine, 164 Worldcup-ro, Yeongtong-gu, Suwon 16499, Republic of Korea

## Abstract

Liver X receptors (LXRs) have emerged as important regulators of inflammatory gene expression. Previously, we had reported that an LXR*α* gene promoter polymorphism (-1830 T > C) is associated with systemic lupus erythematosus (SLE). Therefore, we assessed cytokine expression in relation to LXR*α* polymorphism in monocyte-derived macrophages from patients with SLE. Macrophages were obtained after 72 hours of culture of human monocytes supplemented with phorbol 12-myristate 13-acetate. Cells were transfected with LXR*α* promoter constructs. Additionally, peripheral blood mononuclear cell- (PBMC-) derived macrophages from the patients were evaluated for proinflammatory cytokines in relation to the genotypes of LXR*α* -1830 T > C. The expression of LXR*α* was increased in macrophages; levels of proinflammatory cytokines were decreased with LXR*α* expression. Production of proinflammatory cytokines varied depending on LXR*α* -1830 T > C genotype. In particular, expression of LXR*α* was decreased and that of proinflammatory cytokines was increased for LXR*α* -1830 TC genotype compared to that for TT genotype. The data were consistent in PBMC-derived macrophages from patients with SLE. Increased proinflammatory cytokines is related to TLR7 and TLR9 expression. These data suggest that the expression levels of LXR*α*, according to LXR*α* -1830 T > C genotype, may contribute to the inflammatory response by induction of inflammatory cytokines in SLE.

## 1. Introduction

Liver X receptors (LXRs) were originally identified as ligand-dependent transcriptional activators that induce target genes involved in lipid metabolism. The subfamily consists of two isoforms: LXR*α* and LXR*β*. Gene transcription is modulated by LXRs, which heterodimerize with the retinoid X receptor and bind to LXR-response elements in the transcriptional regulatory regions of their target genes [1]. Recently, LXRs have been reported to regulate macrophage inflammatory responses, phagocytosis, and apoptosis [2, 3]. LXRs inhibit the transcription of proinflammatory cytokines such as interleukin-1*β* (IL-1*β*), IL-6, and tumor necrosis factor-*α* (TNF-*α*) via their promoters or enhancers [3]. One study showed that LXRs mediate the regulation of Th17 cell differentiation and autoimmunity [4]. Furthermore, LXR has been demonstrated to be involved in the upregulation of various genes, including apoptotic inhibitor of macrophage and arginase II [5]. Therefore, LXRs have emerged as important regulators of inflammatory gene expression in several inflammatory diseases [6–8].

Systemic lupus erythematosus (SLE) is a chronic autoimmune disease with various clinical manifestations and autoimmune serologic markers. The pathogenesis of SLE is unclear, but several causes, such as genetic background, environmental factors, and disturbance in both innate and adaptive immunity, have been proposed as contributing factors for the development of the disease [9]. Disturbances in apoptotic cell clearance, hyperactive immune cells, and an abnormal production of autoantibodies are observed as major pathological features of SLE [9, 10]. In particular, uncleared apoptotic cells and their accumulation in tissues have been suggested to contribute most to the inflammation in SLE [11]. Some molecules, such as growth arrest-specific 6 and protein S, enhance the recognition and susceptibility of apoptotic cells to phagocytosis [12, 13]. These interact with receptor tyrosine kinases of the TAM (Tyro-3, Axl, and Mer) family [14]. The loss of regulation of inflammation and delayed clearance of apoptotic materials are associated with the development of a lupus-like syndrome in TAM knockout mice [15]. In particular, Mer signaling has been reported to increase the transcriptional activity of LXR to promote the resolution of acute sterile inflammation [16]. Therefore, LXRs might play an important role in the regulation of inflammatory gene expression in SLE. However, the association between LXR activation or expression and pathogenesis of SLE has not been well addressed.

We had previously reported that LXR*α* gene (*NR1H3*) promoter polymorphisms are associated with SLE in Koreans [17]. Specifically, the -1830 T > C polymorphism within *NR1H3* promoter region was associated with clinical manifestations of SLE; increased B cell proliferation and decreased *NR1H3* mRNA expression were observed in patients with -1830 TC genotype compared to those with the -1830 TT genotype. Therefore, in this study, we assessed cytokine expression in different LXR*α* polymorphism in monocyte-derived macrophages from patients with SLE. Furthermore, we evaluated the effect of LXR activation on proinflammatory cytokine secretion induced by several Toll-like receptor (TLR) agonists.

## 2. Materials and Methods

### 2.1. Cell Culture

U937 cells (human myelomonocytic leukemia cell line) were cultured in RPMI 1640 medium supplemented with 10% heat-inactivated fetal bovine serum (FBS) at 37°C in a 5% CO_2_ incubator. THP-1 cells (human acute monocytic leukemia cell line) were cultured in RPMI 1640 medium supplemented with 10% heat-inactivated FBS and 0.05 mM 2-mercaptoethanol at 37°C in a 5% CO_2_ incubator. Macrophages were obtained after 72 h of culture of human monocytes (U937 or THP-1) in RPMI 1640 medium (Gibco by Life Technologies, Grand Island, NY) supplemented with PMA (40 nM or 80 nM). Cells were cultured at a density of 1 × 10^6^ cells/mL in 24-well plates (Corning, NY), and the cells were transfected with 1 *μ*g control pGL3 or LXR*α* promoter constructs, using FuGENE HD (Promega, Madison, WI), Lipofectamine 2000 (Thermo scientific, Fremont, CA), and ultra TRAX transfection agent (GeneDireX, Taoyuan, Taiwan) according to the manufacturer's instructions. After incubation for 6 h, the medium was replenished with 500 *μ*L of fresh medium with 20% FBS, and the cells were incubated for another 18 h at 37°C in a 5% CO_2_ incubator. Twenty-four hours after transfection, cells were preincubated with LXR agonist (3 *μ*M GW3965 or 5 *μ*M T0901317) at the indicated concentrations for 24 h prior to the addition of TLR ligands: 100 ng/mL ultrapure lipopolysaccharide (LPS; Calbiochem, San Diego, CA), 1 *μ*g/mL CL097 (tlrl-c97, InvivoGen, San Diego, CA), and 1 *μ*M ODN TTAGGG (tlrl-ttag151, InvivoGen) for 24 h.

### 2.2. Ex Vivo Cell Culture

Twelve patients with SLE, who were involved in the previous study, were enrolled again [17]. Among them, 6 patients had LXR*α* -1830 TT and 6 patients had TC genotype. All patients satisfied at least four of the criteria laid out by 1982 revised American College of Rheumatology criteria for SLE [18]. Supplementary Table 1 shows the clinical characteristics and laboratory findings of enrolled 12 SLE patients. This study was approved by the Institutional Review Board of Ajou University Hospital (IRB No. AJIRB-BMR-EXP-14-186). Informed consent was obtained from all subjects. All experiments were performed in accordance with relevant guidelines and regulations.

PBMCs from buffy coats of patients were isolated using Ficoll-Paque PLUS gradient (GE Healthcare Life Sciences, Pittsburgh, PA). The purity of CD14^+^ cells was >90%, as assessed by flow cytometry. CD14^+^ cells were cultured for 5 days at 1 × 10^6^ cells/mL in 6-well plates containing serum-free DMEM media (Gibco, Carlsbad, CA) in the presence of M-CSF (100 ng/mL; R&D Systems, Minneapolis, MN).

LXR agonist, on day 2, was coincubated with either activators or inhibitors of TLR7 and TLR9 for 24 h. Cells were then harvested by centrifugation. Supernatants were collected and immediately stored at -20°C before being tested by enzyme-linked immunosorbent assay (ELISA). Pellets were resuspended in phosphate-buffered saline (PBS), and proteins were extracted for western blot analysis.

### 2.3. Preparation of Plasmid DNA and Transfection

Structures, composed of the LXR*α* -1830 T > C sequence, were assembled carrying each allele. A 500 bp fragment (from -2121 to -1622) of the LXR*α* gene was PCR-amplified using either -1830 T homozygous or -1830 C homozygous genomic DNA as a template and the following primers: forward primer: 5′-CGGCGG**GGTACC**ACATCTATGCCAGCCCTGTTTCAG-3′ (the bold characters represent the KpnI site); reverse primer: 5′-CCGCCG**CTCGAG**ACTGAGCCCCAGCGGCTTTC-3′ (the bold characters denote the XhoI site). Each PCR product was subcloned separately into the KpnI-XhoI site of the pGL3-Basic luciferase reporter vector (Promega, Madison, WI).

### 2.4. RNA Extraction and Quantitative Real-Time PCR

Total RNA was extracted from cells, using an RNeasy Mini kit according to the manufacturer's instruction (Qiagen, Valencia, CA); cDNA was synthesized from total RNA using GoScript Reverse Transcription System kit (Promega, Madison, WI) and 18-residue oligo (dT) (Bioneer, Seoul, Korea). After annealing at 25°C for 5 min and extension at 70°C for 15 min, the product was stored at -20°C until use. The real-time PCR amplification was performed using a Rotor-Gene SYBR Green PCR kit (Qiagen, Valencia, CA). The following PCR conditions were used: heating to 95°C for 5 min, then 40 cycles of 95°C for 5 s, 58°C for 10 s, and 72°C for 30 s. The primers used were as follows: human LXR*α* (F): 5′-AGGGCTGCAAGGGATTCTTCC-3′, (R): 5′-TCTGACAGCACACACTCCTCCC-3′, TNF-*α* (F): 5′-TGCCTATGTCTCAGCCTCTTC-3′, (R): 5′-GGGCCATAGAACTGATGAGAG-3′, IL-1*β* (F): 5′- TCCCAGACAACCACCTTCTC-3′, (R): 5′- ACTGGGCAGACTCAAATTCC -3′, IL-6 (F): 5′- TCCTCATTCCCTCAACTTGG-3′, (R): 5′- GTCAGCAGGCTGGCATTT-3′, IL-10 (F): 5′-TTA CCT GGA GGA GGT GAT GC-3′, (R): 5′-TGG GGG TTG AGG TAT CAG AG-3′, ATP binding cassette 1 (ABCA1) (F): 5′-GAACTGGCTGTGTTCCATGAT-3′, (R): 5′-GATGAGCCAGACTTCTGTTGC-3′, *β*-actin (F): 5′-CAAGAGATGGCCACGGCTGC-3′, (R) 5′-TCCTCTGCATCCTGTCGGC-3′.

### 2.5. Total Protein Extracts and Immunoblot Analysis

Total protein extracts were prepared as described [17]. Equal amounts of protein were resolved by SDS-PAGE and analyzed with anti-LXR*α* (1 : 1,000, ab135039, Abcam, Cambridge, MA), anti-TNF-*α* (1 : 1,000, ab183896, Abcam), anti-interferon-*γ* (IFN-*γ*; 1 : 1,000, EPR1108, Abcam), anti-ABCA1 (PA1-16789, Thermo scientific, Fremont, CA), and anticytoskeletal actin (1 : 10,000, A300-491A, Bethyl Laboratories, Montgomery, TX) antibodies. The secondary antibody used with each was goat anti-mouse antibody (1 : 2,000, AbFrontier, Seoul, Korea), except for anticytoskeletal actin (1 : 20,000). Following transfer and blotting, the proteins of interest were visualized by enhanced chemiluminescence (Pierce, Rockford, IL) and analyzed.

### 2.6. Measurement of Cytokine Production

The concentration of cytokines in cell culture supernatants was analyzed by commercial ELISA, specific for human IL-1*β*/IL-1F2 (DY201-05, R&D, Minneapolis, MN), human TNF-*α* (DY210-05, R&D), human IFN-*γ* (DY285-05, R&D), and human COX-2 (DYC4198-2, R&D) according to the manufacturer's protocol.

### 2.7. Data Analysis

Statistical analyses were performed using the IBM SPSS software ver. 23.0 (IBM Corp., Armonk, NY). The data are shown as mean ± standard deviation (SD) or median and interquartile range, as appropriate. Differences in cytokine levels and LXR*α* levels were determined by Student's *t*-test. A *p* value < 0.05 was regarded as indicator for statistical significance.

## 3. Results

### 3.1. Monocyte-Derived Macrophage Differentiation Upregulates LXR*α* Expression whereas LXR Agonists Downregulate Proinflammatory Cytokines in Monocyte-Derived Macrophages

**Figure 1:**
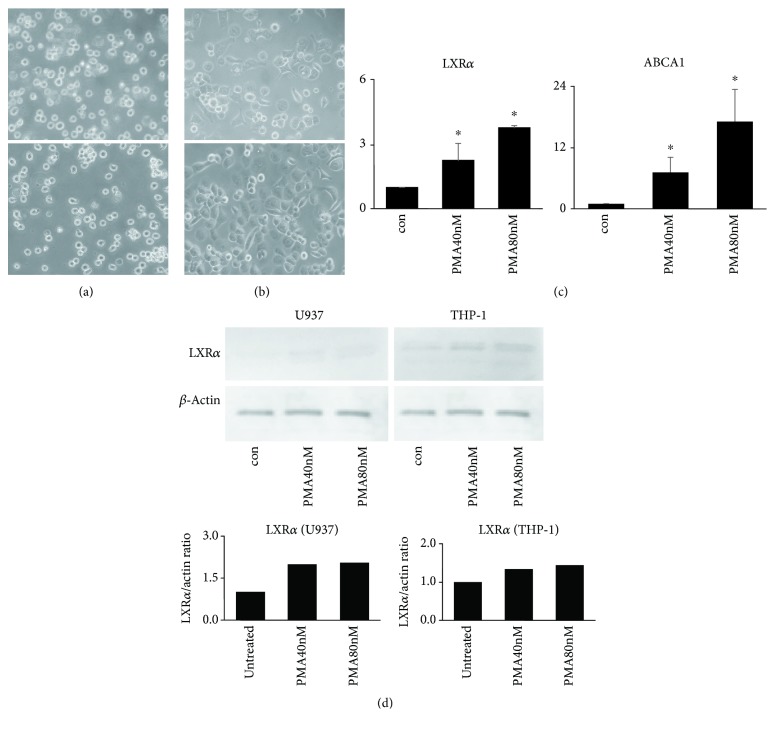
Monocyte-derived macrophage differentiation upregulates liver X receptor *α* (LXR*α*) expression. (a) Untreated THP-1 cells (A) and THP-1 cells treated with PMA for 72 h (B). (b) Untreated U937 cells (A) and PMA-treated U937 cells after 72 h (B). (c) mRNA expression of LXR*α* and ABCA1 is increased in monocyte-derived macrophages after treatment with PMA for 72 h. (d) Protein levels of LXR*α* are increased in monocyte-derived macrophages (THP-1 and U937) after treatment with PMA for 72 h. For immunoblot analysis of LXR*α*, total cellular proteins were extracted from THP-1 and U937 cells treated with PMA. Data are shown from three independent experiments. Values are the means and SD. ^∗^ *p* ≤ 0.05 vs. controls.

To confirm the expression of LXR*α* in monocyte-derived macrophages, we measured mRNA and protein abundance of LXR*α* and LXR target gene *ABCA1* for 72 h after phorbol 12-myristate 13-acetate (PMA) treatment in U937 and THP-1 cells. Monocytes (U937 and THP-1 cells) were differentiated to macrophages after 72 h of PMA treatment (Figures 1(a) and 1(b)).  Figure 1(c) shows a significant increase in LXR*α* and *ABCA1* mRNA abundance after macrophage differentiation with PMA. The data for protein levels were consistent with those of mRNA levels (Figure 1(d)).

**Figure 2:**
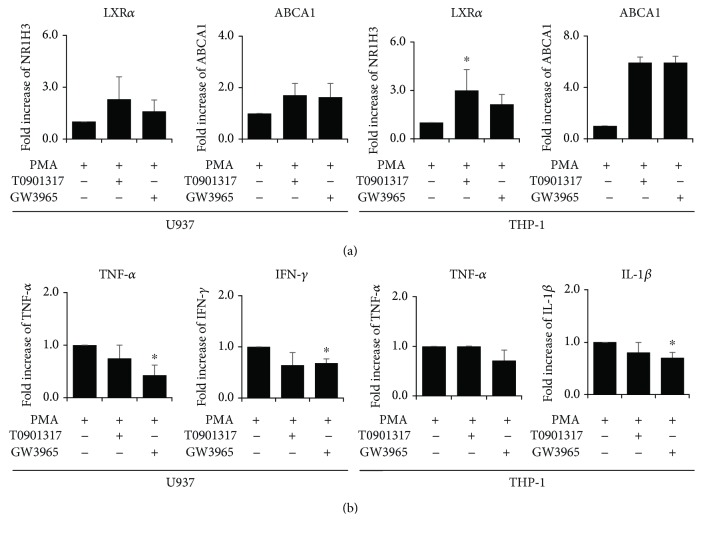
Liver X receptor (LXR) agonists increase LXR*α* and decrease proinflammatory cytokine levels. (a) mRNA expression of LXR*α* and ABCA1 was increased in monocyte-derived macrophages treated with LXR agonist (T0901317 or GW3965) after treatment with 40 nM of PMA for 72 h. (b) mRNA expression of tumor necrosis factor-*α* (TNF-*α*), interferon-*γ* (IFN-*γ*), and interleukin-1*β* (IL-1*β*) are shown in monocyte-derived macrophages treated with LXR agonist (T0901317 or GW3965) after treatment with PMA for 72 h. Data are shown from three independent experiments. Values are the means and SD. ^∗^ *p* ≤ 0.05 vs. controls.

To determine whether an LXR*α* agonist would influence LXR*α* and *ABCA1* expression in monocyte-derived macrophages, we treated the cells with LXR agonists, T0901317 and GW3965, for 24 h.  Figure 2(a) shows an increase in LXR*α* and *ABCA1* mRNA abundance upon treatment of macrophages with LXR agonists, although statistically significant difference was found only with the treatment of T0901317 in THP-1 cells. Furthermore, LXR agonists decreased mRNA expression of proinflammatory cytokines such as TNF-*α*, IFN-*γ*, IL-1*β*, and IFN-*α*, however that was significantly different only in TNF-*α* and IFN-*γ* in GW3965-treated U937 cells and in IL-1*β* in GW3965-treated THP-1 cells (Figure 2(b)).

### 3.2. Expression of LXR*α* and Proinflammatory Cytokines in Human Monocyte-Derived Macrophages according to Genotype (-1830 T > C)

**Figure 3:**
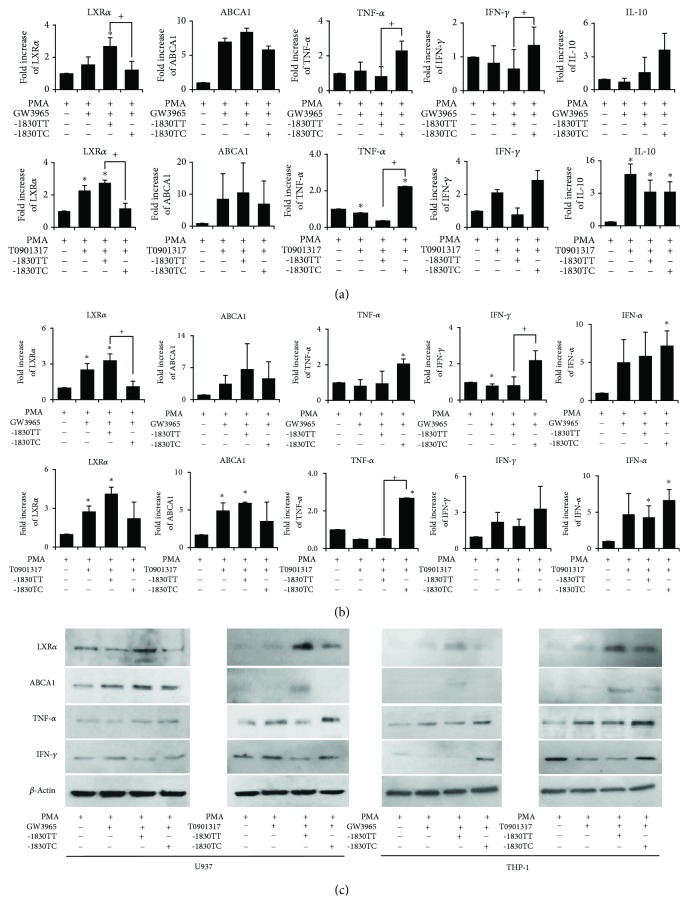
Expression of liver X receptor *α* (LXR*α*) and proinflammatory cytokines in human monocyte-derived macrophages (THP-1 and U937) according to LXR*α* promoter genotypes (-1830 T > C). (a) and (b) mRNA expression of LXR*α* and ABCA1 was increased in monocyte-derived macrophages (THP-1 (a) and U937 (b)) treated with LXR agonist (T0901317 or GW3965) after treatment with PMA for 72 h, and differential expression is shown according to genotype. mRNA expression of tumor necrosis factor-*α* (TNF-*α*), interferon-*γ* (IFN-*γ*), IFN-*α*, and interleukin-10 (IL-10) was shown in monocyte-derived macrophages treated with LXR agonists after treatment with PMA for 72 h according to LXR*α* promoter genotypes. (c) Protein expression levels of LXR*α*, ABCA1, TNF-*α*, and IFN-*γ* are shown for monocyte-derived macrophages treated with LXR agonist. For immunoblot analysis of LXR*α*, ABCA1, TNF-*α*, and IFN-*γ*, total cellular proteins were extracted from THP-1 or U937 derived macrophages treated with LXR agonist. Data are shown from three independent experiments. Values are the means and SD. ^∗^ *p* ≤ 0.05 vs. controls.

To verify the involvement of LXR*α* -1830 T > C genotype in the expression of LXR*α* and proinflammatory cytokines in human monocyte-derived macrophages, we evaluated the endogenous LXR*α* -1830 T > C genotype in monocyte cells (U937 and THP-1 cells). We confirmed apriori that the genotypes of U937 and THP-1 cells were TT. The reporter constructs with the promoter sequence carrying each allele (-1830 T > C) were transfected into U937 or THP-1 cell lines (Supplementary Figure 1). The expression of LXR*α* in the monocytes transfected with pGL3-Basic vector only (control), TT type vector, and TC type vector were not different (data not shown). The transfected monocytes were differentiated to macrophages after 72 h of PMA treatment, and the cells were treated with T0901317 or GW3965 for 24 h. We confirmed that mRNA expression of LXR*α* and *ABCA1* between 24 h and 72 h treatment of PMA was similar and was at its best after treatment with LXR agonists at 24 h (data not shown). The mRNA expression of LXR*α* and *ABCA1* was decreased in TC genotype-transfected U937 cells compared to that in TT genotype-transfected cells (Figure 3(a)). Further, mRNA expressions of proinflammatory cytokines, including TNF-*α* and IFN-*γ*, were increased in TC genotype-transfected U937 cells compared to that in TT genotype-transfected cells. The data for mRNA levels of LXR and proinflammatory cytokines in transfected THP-1 cells were similar to those in the U937 cells (Figure 3(b)); the protein levels were consistent with the measured mRNA levels (Figure 3(c)). The protein levels of LXR*α* and ABCA1 were decreased in TC genotype-transfected U937 cells compared to that in TT genotype-transfected cells. Further, the protein expression of TNF-*α* and IFN-*γ* was increased in TC genotype-transfected U937 cells compared to that in TT genotype-transfected cells. The corresponding data in transfected THP-1 cells were similar to those in the U937 cells.

### 3.3. Effect of LXR Activation on Proinflammatory Cytokine Production in Human TLR-Stimulated Monocyte-Derived Macrophages

**Figure 4:**
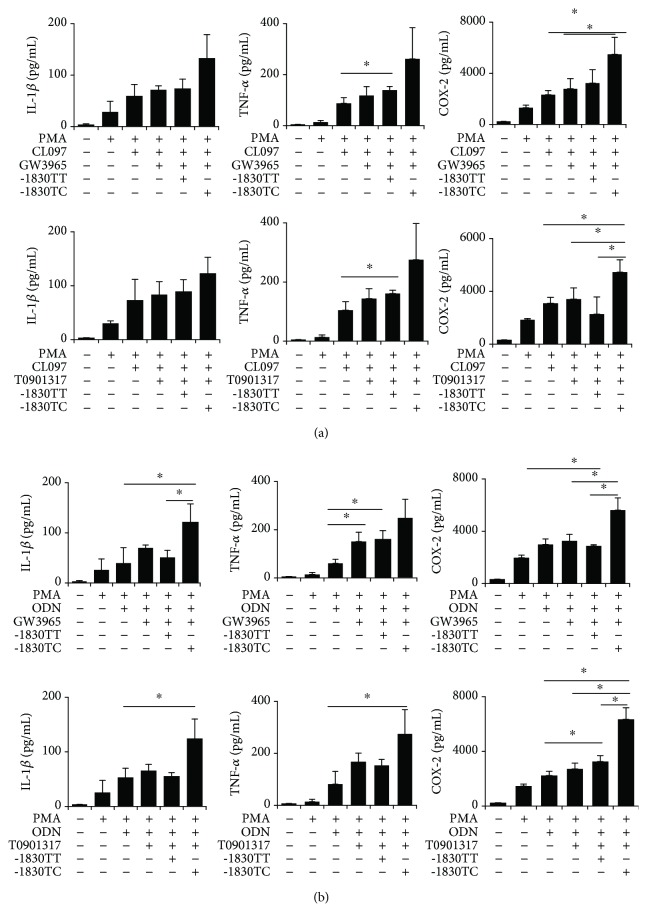
Liver X receptor (LXR) activation in proinflammatory cytokine secretion in human toll-like receptor- (TLR-) stimulated macrophages according to LXR*α* promoter genotypes. PMA-treated U937 cells transfected with LXR*α* -1830 TT or TC genotype were cultured in the presence of LXR agonist (T0901317 or GW3965) for 24 h prior to stimulation with TLR ligands CL097 (TLR7/8, (a)) and ODN (TLR9, (b)). mRNA expression of several cytokines was measured by quantitative real-time PCR. Data are shown from three independent experiments. Values are the means and SD. ^∗^ *p* ≤ 0.05 vs. controls.

TLRs have been widely implicated as the pathogenic drivers in SLE, and TLR2, TLR4, TLR7, and TLR9 have been shown to be expressed at higher levels in B cells, peripheral blood mononuclear cells (PBMC), or kidney tissues [19–22]. Moreover, LXR agonists have been shown to regulate TLR-induced macrophage cytokine secretion with TLR agonists [23–26]. Therefore, we evaluated the effect of LXR activation on proinflammatory cytokine secretion induced by several TLR agonists. PMA-treated U937 cells were cultured in the presence of T0901317 or GW3965 for 24 h prior to stimulation with TLR ligands LPS (for TLR4), CL097 (for TLR7/8), and ODN (for TLR9). The proinflammatory cytokines were not altered in the macrophages stimulated with LPS in the presence of LXR agonists. Further, secretion of proinflammatory cytokines was slightly different in the macrophages stimulated with CL097 in the presence of LXR agonists, though not statistically significant (Figure 4(a)). The secretions were significantly augmented for only TNF-*α* in macrophages stimulated with ODN in the presence of LXR agonists (Figure 4(b)).

**Figure 5:**
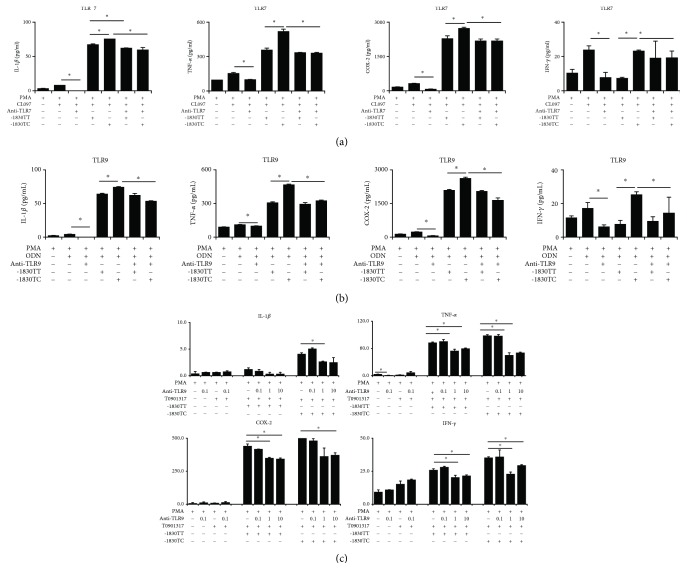
Toll-like receptor 7 (TLR7) and TLR9 inhibitors attenuate proinflammatory cytokine responses by liver X receptor (LXR) agonists in human TLR-stimulated monocyte-derived macrophages, especially in LXR*α* -1830 TC genotype-transfected U937 cells. mRNA expression of LXR*α*, ABCA1, and several cytokines was measured by quantitative real-time PCR. (a) Treatment with TLR7 inhibitors after stimulating with TLR ligands CL097 (TLR7/8). (b) Treatment with TLR9 inhibitors after stimulating with TLR ligands ODN (TLR9). (c) Dose response of TLR9 inhibitors in the presence of LXR agonist (T0901317 or GW3965) for 24 hrs. Data are shown from three independent experiments. Values are the means and SD. ^∗^ *p* ≤ 0.05 vs. controls. The data obtained upon treatment of the cells with TLR9 inhibitor were similar to those obtained for the TLR7 inhibitor (Figure 5(b)). The TLR9 inhibitor decreased the levels of IL-1*β*, TNF-*α*, COX-2, and IFN-*γ* in U937 cells treated with PMA for 24 h prior to stimulation with TLR ligand ODN. The levels of proinflammatory cytokines were increased in both TC and TT genotype-transfected cells, stimulated with ODN, but especially increased in TC genotype-transfected U937 cells compared to those in TT genotype-transfected macrophages. Furthermore, treatment with TLR9 inhibitor (more than 1 *μ*M) significantly decreased the levels of IL-1*β*, TNF-*α*, COX-2, and IFN-*γ* in TC genotype-transfected U937 cells stimulated with LXR ligand (T0901317), and those of TNF-*α*, COX-2, and IFN-*γ* in TT genotype-transfected U937 cells (Figure 5(c)).

To verify the involvement of LXR*α* -1830 T > C genotype in the expression of proinflammatory cytokines in monocyte-derived macrophages treated with TLR agonists, U937 cells were transfected with reporter constructs harboring either of the genotypes. Interestingly, the levels of TNF-*α* and cyclooxygenase-2 (COX-2) were increased in TC genotype-transfected U937 cells compared to those in TT genotype-transfected macrophages stimulated with CL097 (Figure 5(a)). Furthermore, the levels of IL-1*β*, TNF-*α*, and COX-2 were increased in TC genotype-transfected U937 cells compared to those in TT genotype-transfected macrophages stimulated with ODN (Figure 4(b)).

### 3.4. TLR7 And TLR9 Inhibitors Attenuate Proinflammatory Cytokine Production in TLR-Stimulated Human Monocyte-Derived Macrophages, Especially in TC Genotype-Transfected U937 Cells

The levels of proinflammatory cytokines were increased in both TC and TT genotype-transfected cells stimulated with CL097, but relatively more in TC genotype-transfected U937 cells compared to those in TT genotype-transfected macrophages. Treatment with TLR7 inhibitor significantly decreased the levels of IL-1*β*, TNF-*α*, COX-2, and IFN-*γ* in TC genotype-transfected CL097-stimulated cells, whereas only the level of IL-1*β* was decreased in TT genotype-transfected U937 cells (Figure 5(a)).

The data obtained upon treatment of the cells with TLR9 inhibitor were similar to those obtained for the TLR7 inhibitor (Figure 5(b)). The TLR9 inhibitor decreased the levels of IL-1*β*, TNF-*α*, COX-2, and IFN-*γ* in U937 cells treated with PMA for 24 h prior to stimulation with TLR ligand ODN. The levels of proinflammatory cytokines were increased in both TC and TT genotype-transfected cells, stimulated with ODN, but especially increased in TC genotype-transfected U937 cells compared to those in TT genotype-transfected macrophages. Furthermore, treatment with TLR9 inhibitor (more than 1 *μ*M) significantly decreased the levels of IL-1*β*, TNF-*α*, COX-2, and IFN-*γ* in TC genotype-transfected U937 cells stimulated with LXR ligand (T0901317), and those of TNF-*α*, COX-2, and IFN-*γ* in TT genotype-transfected U937 cells (Figure 5(c)).

### 3.5. Expression of Proinflammatory Cytokines, Based on LXR*α* -1830 T > C Genotype, in LXR Agonist-Treated PBMC-Derived Macrophages of Patients with SLE

**Figure 6:**
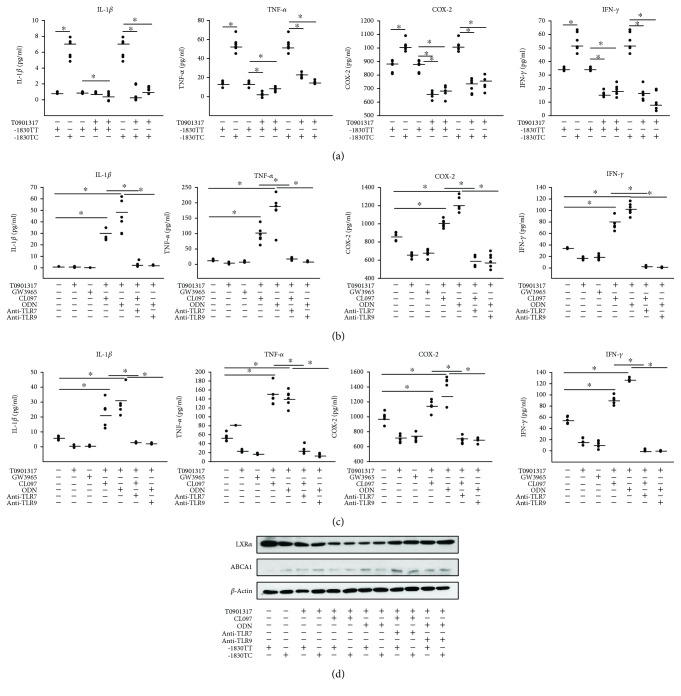
Expression of proinflammatory cytokines after treatment with liver X receptor (LXR) agonist in peripheral blood mononuclear cell- (PBMC-) derived macrophages of patients with SLE according to the *LXRα* -1830 T > C genotypes. mRNA expression of interleukin-1*β* (IL-1*β*), tumor necrosis factor-*α* (TNF-*α*), cyclooxygenase-2 (COX-2), and interferon-*γ* (IFN-*γ*) was measured by quantitative real-time PCR. (a) Treatment with LXR agonists. (b) and (c) Treatment with TLR7 or TLR9 inhibitors and LXR agonists in human TLR7/8 agonist- (CL097-) or TLR9- (ODN-) stimulated PBMC-derived macrophages from SLE patients with the *LXRα* -1830 TT (b) and TC (c) genotype. (d) Protein levels of LXR*α* and ABCA1 according to the *LXRα* -1830 TT and TC genotype after treatment with TLR7 or TLR9 inhibitors. For immunoblot analysis of LXR*α* and ABCA1, total cellular proteins were extracted from PBMCs of SLE patients with the genotypes -1830 TT or TC. Data are shown from three independent experiments. Values are the means and SD. ^∗^ *p* ≤ 0.05 vs. controls.

The PBMC from patients with SLE were differentiated to macrophages after treatment with macrophage colony-stimulating factor (M-CSF) for 72 h, which were then treated with T0901317 and GW3965 for 24 h. The data for the levels of proinflammatory cytokines in PBMC-derived macrophages of patients, according to the *LXRα* -1830 T > C genotypes, were similar to those for the macrophage cell lines transfected with reporter constructs harboring either of the genotypes (Figure 6(a)). The basal levels of proinflammatory cytokines including IL-1*β*, TNF-*α*, COX-2, and IFN-*γ* were increased in patients with TC genotype compared to those in patients with TT genotype. After treatment with LXR agonists, the level of proinflammatory cytokines in macrophages was decreased for both TT and TC genotypes in PBMCs of patients with SLE, but more strikingly so in TC genotype individuals.

TLR7 and TLR9 ligands augment proinflammatory cytokines in LXR agonist-treated macrophages derived from PBMCs of patients with SLE (harboring LXR*α* -1830 T > C genotypes; Figures 6(b) and 6(c)). The TLR7 inhibitor decreased the levels of IL-1*β*, TNF-*α*, COX-2, and IFN-*γ* in macrophages treated with M-CSF for 24 h prior to stimulation with LXR agonist (T0901317) and CL097 (TLR7/8) in both LXR*α* -1830 TT and TC genotypes. Also, treatment with TLR9 inhibitor significantly decreased the levels of IL-1*β*, TNF-*α*, COX-2, and IFN-*γ* in macrophages stimulated with LXR agonist (T0901317) and ODN (TLR9) in both genotypes. Furthermore, immunoblot analysis was performed with antibodies specific to LXR*α* and ABCA1 in M-CSF-treated macrophages with TLR ligands (Figure 6(d)). TLR7 and TLR9 ligands decreased LXR*α* and ABCA1 levels, and treatment with TLR7 or TLR9 inhibitors could recover the levels.

## 4. Discussion

In this study, we showed that the expression of LXR*α* is increased in human monocyte-derived macrophages compared to that in unstimulated monocytes. Levels of proinflammatory cytokines, such as IL-1*β* and TNF-*α*, are decreased with increased expression of LXR*α*. Interestingly, the production of proinflammatory cytokines depends on the expression of LXR*α* -1830 T > C genotypes, consistent with our previous findings [17]. Decreased LXR*α* expression with increased proinflammatory cytokine expression was observed in human monocyte-derived macrophages transfected with the TC genotype of LXR*α* -1830 T > C compared to that in cells transfected with the TT type. These data are consistent in human PBMC-derived macrophages, isolated from patients with SLE, according to respective genotype. Furthermore, increased expression of proinflammatory cytokines in the TC genotype of LXR*α* -1830 is related to TLR7 and TLR9 expression.

An important role of macrophages, in the control of inflammation, is the removal of dying cells [27]. This function could be induced through nuclear receptors, such as PPAR*γ*, PPAR*δ*, and LXR; activation of these signaling pathways could suppress inflammation. Several studies showed that LXR activation exhibited potent anti-inflammatory activities [8, 28–31]. A previous study evaluated the effect of LXR agonists on innate immunity responses in human primary lung macrophages and in a preclinical rodent model of lung inflammation [31]. The authors demonstrated an LXR-dependent reduction in lung neutrophils in a rodent model of lung inflammation. However, this inhibition was not associated with suppression of NF-*κβ*/AP-1 DNA binding. Therefore, these results suggest that anti-inflammatory activity of LXR agonists was not via inhibition of the NF-*κ*B pathway. A recent study identified the lipid transporter ABCA1 as a critical mediator for anti-inflammatory effects of LXR [30]. The activation of LXR inhibited the signaling pathway from TLR2, 4, and 9 to their downstream NF-*κ*B and MAPK effectors through ABCA1-dependent changes. In the present study, we showed the anti-inflammatory effects of LXR in human monocyte-derived macrophages. The expression of LXR*α* was increased in human monocyte-derived macrophages while levels of proinflammatory cytokines were decreased with LXR*α* expression, consistent with previous reports [8, 28–31].

However, LXR activation is known to potentiate proinflammatory cytokine secretion in LPS-activated human macrophages, and this is suggested to be related to increased expression of TLR4 [24, 32, 33]. A recent study showed that LXR activation leads to a dramatic increase in proinflammatory cytokine secretion driven by TLR1/2, TLR2/6, and TLR7/8 [24]. The authors showed that the LXR pathway is upregulated in rheumatoid arthritis synovial macrophages and activation of LXRs by ligands in synovial fluid augments TLR-driven cytokine secretion. However, the data for LXR activation, in the present study, with TLR agonists was not similar to that. Although proinflammatory cytokines were not augmented in macrophages stimulated with several TLR receptor ligands in the presence of LXR agonists, only the secretion of TNF-*α* was elevated in macrophages stimulated with TLR9 ligands in the presence of LXR agonists. Therefore, our results suggest that LXR activation does not potentiate proinflammatory cytokine secretion (except for TNF-*α*) through the upregulation of TLR7 and TLR9 expression in patients with SLE. Interestingly, the inflammatory cytokine response was dependent on the duration of pretreatment with LXR agonist in primary human macrophage; short-term pretreatment reduced the inflammatory response to TLR4 ligand; however, pretreatment longer than 48 h, with LXR agonist, significantly enhanced TLR4 ligand response [33]. Our results could possibly be the outcome of pretreatment with LXR agonist for 24 h. However, further studies would be required to resolve the mechanism by which LXR activation promotes TNF-*α* production by interaction with TLR9.

We previously evaluated the functional effects of the LXR*α* -1830 T > C polymorphism and reported that the -1830 T allele-containing reporter construct had higher promoter activity than that containing the corresponding C allele [17]. Proliferation of B cells of the LXR*α* -1830 TC type was found to have increased beyond those of the TT type or of LXR agonist-treated B cells from patients with SLE. Furthermore, *NR1H3* mRNA expression levels were lower in *NR1H3* -1830 TC type B cells than in TT type cells. To investigate the effects of the *NR1H3*-1830 T > C polymorphism on LXR expression and inflammation in macrophages, we transfected the reporter promoter gene construct, carrying the LXR*α* -1830 TT and TC genotypes, into THP-1 and U937 cells. We also treated cells with T0901317 and GW3965 for confirmation of the effects of LXR agonists. The results were similar to our previous data with B cells. The expression of LXR*α* and ABCA1 was decreased in TC genotype-transfected macrophages compared to that in TT genotype-transfected cells. Further, the expression of proinflammatory cytokines was increased in TC genotype-transfected cells compared to that in the TT genotype-transfected cells. These data are consistent with those of an ex vivo study on patients with SLE. Basal levels of proinflammatory cytokines were higher in macrophages from patients with the TC genotype compared to those with the TT genotype, and similar patterns were identified after treatment with LXR agonists. These data suggest that the TC genotype of LXR*α* -1830 leads to low expression of LXR and insufficient effect of LXR on proinflammatory cytokines compared to that of TT genotype. The low expression of LXR might be associated with susceptibility or disease activity in SLE. Proinflammatory cytokine levels were significantly decreased in TC genotype-transfected macrophages after treatment with TLR7 or TLR9 inhibitors. These data are consistent with those of an *ex vivo* study on PBMCs from patients with SLE with respect to the TC and TT genotypes of LXR*α* -1830. The results suggest that antagonists targeting TLR signaling could be effective for the treatment of SLE, especially in patients with the TC genotype.

## 5. Conclusions

Our results imply that expression of LXR*α* according to LXR*α* -1830 T > C genotypes may contribute to the inflammatory responses by inducing inflammatory cytokines in SLE. In particular, the LXR*α* -1830 TC genotype leads to low expression of LXR and insufficient control on proinflammatory cytokine secretion.

## Data Availability

The data used to support the findings of this study are included within the article.

## Supplementary Materials

Supplementary Materials Supplementary Table 1: clinical characteristics of SLE patients (*n* = 12). Supplementary Figure 1: pGL3-Basic vector circle map and preparations of *LXRα* -1830 T > C reporter constructs. The liver X receptor A (*LXRA)* gene promoter was separately subcloned into a KpnI-XhoI site of pGL3-Basic luciferase reporter gene vector. Additional description: luc+, cDNA encoding the modified firefly luciferase; *Amp^r^*, gene conferring ampicillin resistance in *E. coli*; f1 ori, origin of replication derived from filamentous phage; ori, origin of replication in *E. coli*. Arrows within luc+ and the *Amp^r^* gene indicate the direction of transcription; the arrow in the f1 ori indicates the direction of ssDNA strand synthesis.
