# Supplementary material for: The Liver X Receptor Is Upregulated in Monocyte-Derived Macrophages and Modulates Inflammatory Cytokines Based on LXRα Polymorphism
Source: Mediators Inflamm. 2019 Feb 28;2019:6217548. doi: 10.1155/2019/6217548 (PMC6421810; doi:10.1155/2019/6217548)
Supplement: Supplementary Materials — Supplementary Table 1: clinical characteristics of SLE patients (n = 12). Supplementary Figure 1: pGL3-Basic vector circle map and preparations of LXRα -1830 T > C reporter constructs. The liver X receptor A (LXRA) gene promoter was separately subcloned into a KpnI-XhoI site of pGL3-Basic luciferase reporter gene vector. Additional description: luc+, cDNA encoding the modified firefly luciferase; Ampr, gene conferring ampicillin resistance in E. coli; f1 ori, origin of replication derived from filamentous phage; ori, origin of replication in E. coli. Arrows within luc+ and the Ampr gene indicate the direction of transcription; the arrow in the f1 ori indicates the direction of ssDNA strand synthesis. [file 6217548.f1.pdf]

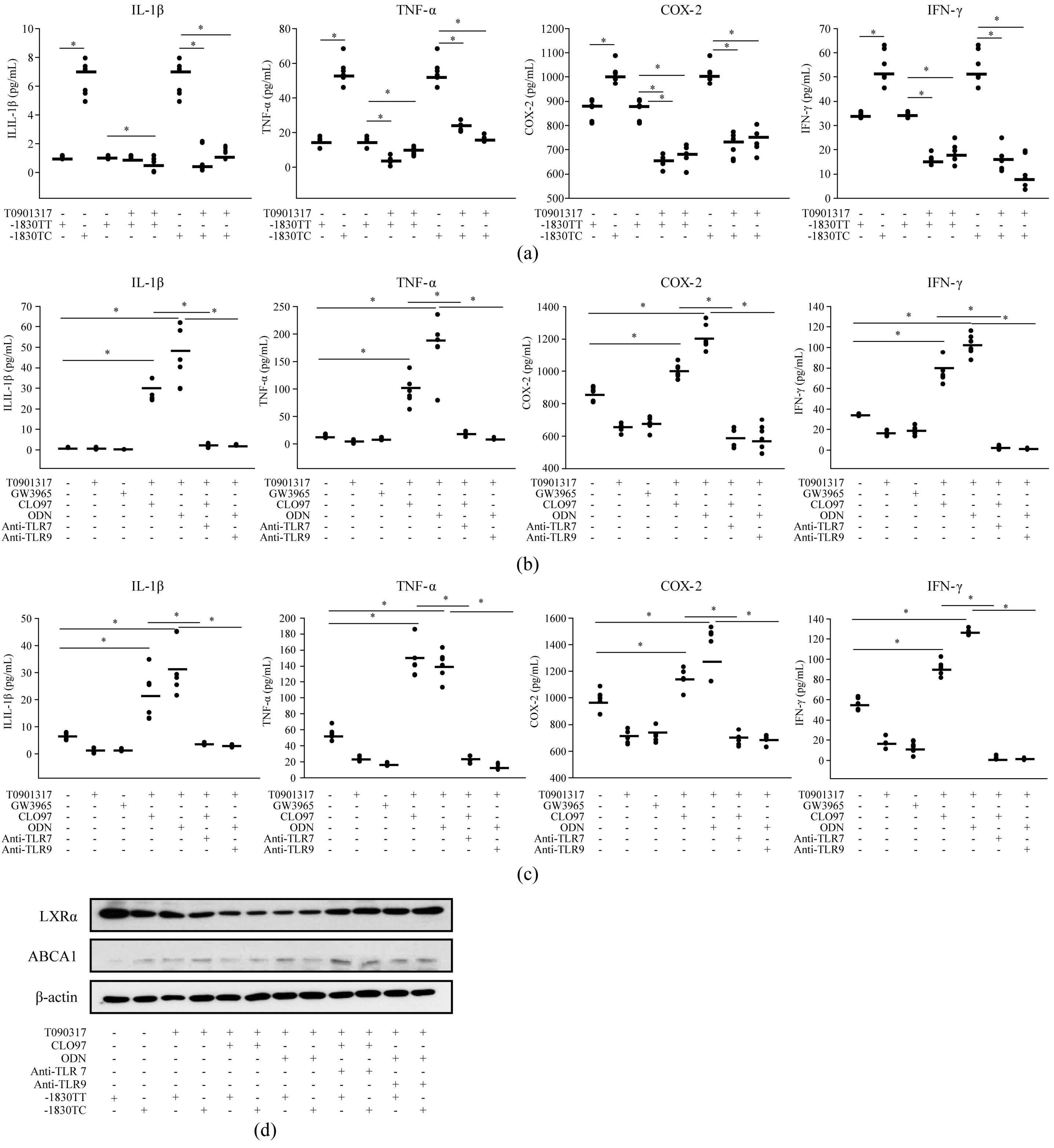
IL-1β
TNF-α
COX-2
IFN-γ
ILIL-1β (pg/mL)
TNF-α (pg/mL)
COX-2 (pg/mL)
IFN-γ (pg/mL)
T0901317
-1830TT
-1830TC
(a)
GW3965
CLO97
ODN
Anti-TLR7
Anti-TLR9
(b)
(c)
LXRα
ABCA1
β-actin
T090317
Anti-TLR 7
(d)

Supplementary Table 1. Clinical characteristics of SLE patients (n=12)

Patient	TT						TC					
	1	2	3	4	5	6	1	2	3	4	5	6
Age	23	24	40	19	48	48	48	49	56	44	27	35
Gender	F	F	F	F	F	F	F	F	F	F	F	F
**Clinical features**												
Fever	-	-	-	-	-	-	-	-	-	-	-	-
Oral ulcer	+	+	+	+	-	-	+	+	+	+	-	+
Arthritis	-	+	-	-	+	+	+	-	+	-	+	-
Rash	+	+	-	+	-	-	-	-	-	+	+	-
Serositis	-	-	-	-	-	-	-	-	-	-	-	-
Nephritis	+	+	+	-	-	-	-	-	-	-	-	+
**Laboratory findings**												
Leukocytes (x 10^3/μL)	7.1	2.2	4.2	3.9	6.7	6.5	4.2	3.5	6.6	3.0	4.8	5.8
Hemoglobin (g/dL)	11.3	10.7	12.7	14.7	12.8	12.2	12.7	13.3	13.4	11.5	12.5	12.8
Platelets (x 10^3/μL)	288	283	212	169	224	256	182	267	217	79	175	208
Anti-dsDNA antibody (IU/mL)	103	103	22.6	3	3	10	6.2	4.6	3	8.3	11.3	92.3
Complement 3, mg/dL	52	35	99	91	72	58	91	104	103	46	82	79
Complement 4, mg/dL	4	3	28	22	23	22	31	29	28	5	13	13
ESR (mm/hr)	20	29	33	4	2	18	43	28	22	6	19	6
CRP (mg/dL)	0.02	0.32	0.08	0.03	0.03	0.08	0.42	0.14	0.19	0.02	0.02	0.09

SLEDAI	14	13	8	4	6	8	6	2	6	10	8	6
**Medication**												
Hydroxychloroquine	+	+	-	+	+	+	+	+	+	-	-	-
Azathioprine	-	+	-	-	-	-	-	+	-	-	+	-
MMF	+	-	-	-	-	-	-	-	-	-	-	+
Prednisolone dose (mg/day)	20	5	1.25	0	1.25	2.5	1.25	0	2.5	5	5	5

SLE, systemic lupus erythematosus; ESR, erythrocyte sedimentation rate; CRP, C-reactive protein; SLEDAI, systemic lupus erythematosus disease activity index; MMF, mycophenolate mofetil.
